# Metamaterial for elastostatic cloaking under thermal gradients

**DOI:** 10.1038/s41598-019-40517-6

**Published:** 2019-03-05

**Authors:** Juan C. Álvarez Hostos, Víctor D. Fachinotti, Ignacio Peralta

**Affiliations:** 1 Centro de Investigación de Métodos Computacionales (CIMEC), Universidad Nacional del Litoral (UNL)/Consejo Nacional de Investigaciones Científicas y Técnicas (CONICET), Predio CCT-CONICET Santa Fe, Ruta 168, Paraje El Pozo, 3000 Santa Fe, Argentina; 2 Department of Chemical Metallurgy, Universidad Central de Venezuela, Los Chaguaramos, 1040 Caracas, Venezuela; 3 Universidad Tecnológica Nacional (UTN)/Facultad Regional Santa Fe, Lavaisse 610, 3000 Santa Fe, Argentina

## Abstract

We introduce the optimization-based method for the design of thermo-mechanical metamaterials and, particularly, for the elastostatic cloaking under thermal loads. It consists of solving a large-scale, nonlinear constrained optimization problem, where the objective function is the error in the cloaking task accomplishment. The design variables define the required metamaterial distribution. In this way, the cloaking task is accomplished, if not exactly, optimally. Further, the design variables dictate how to fabricate the metamaterial, avoiding the uncertainty of simultaneously mimicking several thermal and mechanical effective properties, as required by transformation-based metamaterial design methods.

## Introduction

Recently, the development of metamaterials has allowed the manipulation of temperature and displacement fields in ways inconceivable in nature, including cloaking^1–7^, focusing^1,4,8–12^, inversion^4,12–14^, shielding^15,16^ and channeling^17^ in heat conduction, and cloaking in elastostatics^16,18,19^ and elastodynamics^20–22^.

Classically, metamaterials have been designed following the transformation-optics (TO) approach, which was originally proposed by Leonhardt^23^ and Pendry^24^ for electromagnetic cloaking. This idea was adapted to design most of the aforementioned metamaterials, either for heat flux manipulation^1,3–6,8,9^ or mechanical cloaking^20–22^.

Regarding mechanical problems, Shuvalov^25^ demonstrated that the TO approach produces the anisotropic metamaterials known as Willis materials^26^. In this regard, Milton *et al*.^27^ demonstrated the need to use, besides the standard fourth-order elasticity tensor, third-order tensors for achieving a transformation invariant form of the Navier equations for cloaking purposes, whereas Brun *et al*.^28^ achieved such a transformation without additional third-order tensors but breaking the symmetries of the standard fourth-order elasticity tensor. Additionally, several researchers have made great efforts for the realization of metamaterials issued from TO^18–20,29^. Stenger *et al*.^20^ designed a circular mechanical cloaking device with radially varying effective elastic properties in an attempt to follow the transformed plate equation derived by Farhat *et al*.^30^ Kadic *et al*.^29^ proposed to use pentamodal materials to realize any material from TO. Shortly after, Bückmann *et al*.^18^ made an elastostatic cloaking using pentamodal metamaterials. As an alternative to TO, Bückmann *et al*.^19^ proposed the so-called direct-lattice transformation design approach with application to elastostatic cloaking. Such an approach allows the achievement of realizable metamaterials from lattices. The direct-lattice transformation dictates the geometry of the lattice-type metamaterial, that is, how to fabricate it. On the other hand, the TO defines the inhomogeneous effective material properties required to accomplish a given task, which makes necessary to devise ways to manufacture metamaterials having, or at least mimicking, these particular properties.

Recently, Peralta *et al*.^10^ proposed a metamaterial design method based on mathematical optimization, avoiding coordinate transformations. It consists of solving an optimization problem to minimize the error in the accomplishment of the task assigned to the metamaterial, taking as design variables the parameters that describe the microstructure. Like direct lattice transformation^19^, the optimization-based approach directly prescribes how to fabricate the metamaterial at a point, but it excels the former since not only lattices but any quantitatively characterized material is allowed. It also excels the preceding design methods in terms of versatility since it applies to general heat flux manipulation tasks including concentration^10–12^, shielding^12^ and inversion^12^. Further, it was easily extended to design very easy-to-fabricate devices made of piecewise homogeneous metamaterials^11^ for heat flux concentration, and even standard isotropic materials for heat flux inversion^12^ and elastostatic cloaking^16^.

The optimization-based procedures proposed so far for the design of metamaterials, have been developed in heat conduction^10–12^ and the elastostatic problems^16^. However, an optimization-based procedure for the design of elastostatic metamaterials under thermal gradients involves the coupled solution of the heat transfer and elasticity problems, namely, a thermo-elastic problem. The force equilibrium is then affected by the thermal loads resulting from the temperature variations throughout the material system, which introduces a further complication in the optimization procedure. So, it is worth to mention that the metamaterial design for the accomplishment of a given thermo-mechanical task will be dictated in terms of a proper spatial arrangement of the thermal conductivity, elastic moduli and thermal expansion. In agreement with the aforementioned aspects, in this work we are interested in the design of metamaterials under coupled thermal and mechanical phenomena, which is unprecedented in the literature. To this end, we will take advantage of the optimization-based design approach versatility.

## Methodology

Let Ω be a body made of an arbitrary material, and **u**_0_ be the displacement field in Ω under given thermal and mechanical boundary conditions. When the material in the region Ω_incl_ ∈ Ω is replaced by a material with markedly different thermal conductivity, stiffness and/or thermal expansion, the displacement field in Ω is significantly affected. For the purpose of cloaking, let us define a metamaterial occupying the region Ω_met_ around Ω_incl_ such that the resulting displacement field **u** resembles **u**_0_ in a given region Ω_cloak_ ∈ Ω.

Assuming linear thermoelastic behavior and small strains, the displacement field in Ω is governed by the equilibrium equation

1 $${\rm{div}}\,{\boldsymbol{\sigma }}+\rho {\bf{b}}={\bf{0}},$$

where *ρ* is the density, **b** is the body force, and **σ** is the Cauchy stress tensor defined by

$${\boldsymbol{\sigma }}={\bf{C}}\,:[{\boldsymbol{\varepsilon }}-{\boldsymbol{\alpha }}(T-{T}_{{\rm{ref}}})],$$

being ***ε*** = [grad **u** + (grad **u**)^*T*^]/2 the infinitesimal strain tensor, **C** the elastic moduli tensor, ***α*** the thermal expansion tensor, *T* the temperature and *T*_ref_ the temperature for zero thermal strain. The dependence of **u** on *T* obliges to couple the solution of the equilibrium Eq. (1) to that of the heat conduction equation

2 $${\rm{div}}({\bf{k}}\,{\rm{grad}}\,T)+Q=\mathrm{0,}$$

where **k** is the thermal conductivity tensor and *Q* is the internal heat source.

Here, this coupled thermomechanical problem is solved using the finite element method (FEM), as detailed by Fachinotti *et al*.^31^ With Ω divided in finite elements, let the microstructure at each finite element Ω^(*e*)^ in the non-homogeneous metamaterial region Ω_met_ be quantitatively characterized by the vector **p**^(*e*)^ of microparameters. Consequently, any effective material property at Ω^(*e*)^ is a function of **p**^(*e*)^, as it is currently the case of the thermal conductivity, the thermal expansion and the elastic moduli. To couple the thermal fields to the mechanical response, introduces a further complication in the optimization based design of metamaterials for cloaking purposes. Although the objective function is defined only in terms of the displacement field, the cloaking task fulfillment will be affected by the dependence of both displacement and temperature on the microparameters distribution. The current approach can be seen as a generalization of that proposed by Sigmund and Torquato^32^ for the design of metamaterials with extreme thermal expansion features, where only the mechanical properties were considered, since the temperature distribution was prescribed to be homogeneous irrespectively of the inhomogeneity in thermal conductivity. Note that the problem addressed by Sigmund and Torquato does not involve either the coupling between the force equilibrium and heat conduction equations, or the temperature dependence on the microparameters. On the other hand, such aspects are properly addressed in the fully coupled thermo-mechanical problem proposed in this communication.

Now, with the effective material properties throughout the metamaterial region Ω_met_ characterized by the vector **P** = [**p**^(1)^ **p**^(2)^ …] containing the characteristic microparameters **p**^(*e*)^ of all the elements Ω^(*e*)^ ∈ Ω_met_, the displacement in the whole body Ω depends on **P**, i.e., **u** = **u**(**x**, **P**) for all **x** ∈ Ω; see the work of Fachinotti *et al*.^31^ for details on this dependence when the equilibrium and heat conduction equations are solved using FEM.

Then, the problem of metamaterial design for elastostatic cloaking consists of finding **P** such that **u**(**x**,**P**) = **u**_0_(**x**) for all **x** ∈ Ω_cloak_. Numerically approximated, it can be stated as: to find **P** such that

3 $${\bf{u}}({\bar{{\bf{x}}}}^{(i)},{\bf{P}})={{\bf{u}}}_{0}({\bar{{\bf{x}}}}^{(i)}),\,i=\mathrm{1,}\,\ldots ,\,{N}_{{\rm{check}}},$$

at a series of predefined checkpoints ${\bar{{\bf{x}}}}^{(i)}\in {{\rm{\Omega }}}_{{\rm{cloak}}}$.

**Figure 1:**
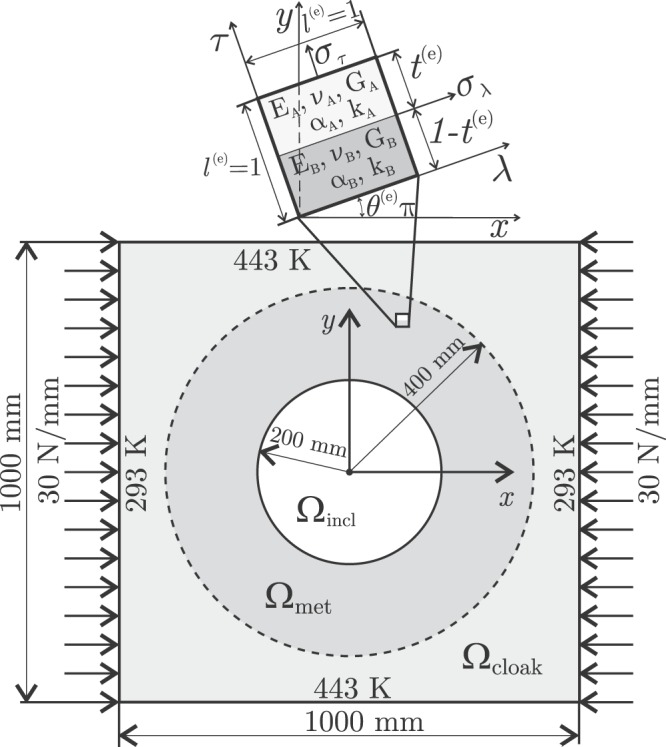
Domain Ω = Ω_cloak_ ∪ Ω_met_ ∪ Ω_incl_ and boundary conditions of the thermo-mechanical problem. Above, the representative volume element (RVE) characterizing the microstructure in the device Ω_met_.

To guarantee the realizability of the microstructure, the search of **P** must be constrained to a feasible design set ${\mathscr{D}}$. In general, this precludes the exact accomplishment of the cloaking task (3) leading to a near-elastostatic cloaking. The near-cloaking problem in elastostatic has also recently been adressed by Craster *et al*.^33^ through a different formulation, which is based on the regularized change of variable originally developed by Kohn *et al*.^34^ for electromagnetic cloaking. In the frame of the optimization based procedure proposed in this communication, let us obtain a design for which the root mean square error (RMSE) in the accomplishment of the task reaches a minimum by solving the nonlinear constrained optimization problem

4 $$\mathop{{\rm{\min }}}\limits_{{\bf{P}}\in {\mathscr{D}}}\sqrt{\frac{1}{{N}_{{\rm{check}}}}\sum _{i=1}^{{N}_{{\rm{check}}}}{\Vert {\bf{u}}({\bar{{\bf{x}}}}^{(i)},{\bf{P}})-{{\bf{u}}}_{0}({\bar{{\bf{x}}}}^{(i)})\Vert }^{2}},$$

where **P** are the design variables. As an application example, let Ω be a square plate made of nylon (See Table 1 for physical properties) under plane strain conditions, free of internal heat sources and body forces, subject to thermal and mechanical boundary conditions as shown in Fig. 1. Assuming a fixed Cartesian reference frame *xyz*, let *z* be the zero-strain direction. After making a circular hole Ω_incl_ at the center of the plate, let us design a metamaterial device occupying the region Ω_met_ (around the hole) such that the displacement field in Ω_cloak_ (outside the device) be affected to a minimum extent.

**Table 1:** Thermal and mechanical properties of nylon, aluminum and polyethylene.

Property	Nylon	Aluminum	Polyethylene
Thermal conductivity	0.31 W(mK)^−1^	*k*_A_ = 205 W(mK)^−1^	*k*_B_ = 0.46 W(mK)^−1^
Young modulus	3 GPa	*E*_A_ = 69 GPa	*E*_B_ = 0.3 GPa
Poisson ratio	0.4	*ν*_A_ = 0.33	*ν*_B_ = 0.46
Shear modulus	1.07 GPa	*G*_A_ = 25.94 GPa	*G*_B_ = 0.103 GPa
Thermal expansion	8 × 10^−5^ K^−1^	*α*_A_ = 2.3 × 10^−5^ K^−1^	*α*_B_ = 15 × 10^−5^ K^−1^

### Parametrization of the microstructure

As metamaterial we choose a laminate of aluminum and polyethylene (Materials A and B in Table 1, respectively), just as depicted in Fig. 1. Although this laminate can be constructed with any pair of materials, it has already been proven that the background material should have intermediate mechanical properties regarding the laminate constituents, in order to guarantee the achievement of an optimal design for cloaking purposes^16^. The microstructure at any finite element Ω^(*e*)^ ∈ Ω_met_ is completely characterized by **p**^(*e*)^ = [*t*^(*e*)^ *θ*^(*e*)^], being *t*^(*e*)^ the relative thickness of aluminum and *θ*^(*e*)^*π* the orientation of the laminate; note that the relative thickness of polyethylene is 1 − *t*^(*e*)^. Although all these materials are isotropic, the aluminum-polyethylene laminate is markedly anisotropic. At each finite element, let us adopt a local Cartesian frame *λτz*, with *τ* normal to the laminate, *λ* and *τ* lying in the *xy* plane, and *z* being the zero-strain direction. The relevant tensorial components of the effective properties referred to the *λτz* frame are listed in Table 2. Analytical expressions for a bilayered laminated under plane stress conditions were obtained by Vasiliev and Morozov^35^ using micromechanical analysis. Following these authors, we derive the current analytical expressions for plane strain conditions. When referred to the local frame *λτz*, all the physical properties of a bilayered laminate depend on the relative thicknesses of the layers, i.e. *t*_A_ and *t*_B_ = 1 − *t*_A_. The dependence on the orientation *θ*^(*e*)^ is introduced when these properties are referred to the Cartesian frame *xyz*. The displacement field **u** is determined by solving the equilibrium equation after the heat conduction equation (that gives the temperature field *T*). Both equations are solved using FEM. Given the symmetry of the domain and boundary conditions, only the quarter of Ω with *x* ≥ 0 and *y* ≥ 0 is modeled using an uniform mesh of 200 × 200 = 40000 bilinear finite elements.

**Table 2:** Effective thermal and mechanical properties of a laminate of materials A and B referred to the local Cartesian frame *λτz*.

Thermal conductivity	*k*_*λλ*_ = *t*_A_*k*_A_ + *t*_B_*k*_B_
	*k*_*ττ*_ = *k*_A_*k*_B_(*t*_A_*k*_B_ + *t*_B_*k*_A_)^−1^
Elastic moduli	${C}_{\lambda \lambda \lambda \lambda }={E}_{\lambda }\mathrm{(1}-{\nu }_{\lambda \tau }{\nu }_{\tau \lambda }\mathrm{)[1}-{\nu }_{\lambda z}^{2}-2{\nu }_{\lambda \tau }{\nu }_{\tau \lambda }\mathrm{(1}+{\nu }_{\lambda z}{)]}^{-1}$
	*C*_*ττττ*_ = *E*_*τ*_(1−*ν*_*λz*_)(1−2*ν*_*λτ*_*ν*_*τλ*_−*ν*_*λz*_)^−1^
	*C*_*λλττ*_ = *E*_*τ*_*ν*_*λτ*_(1−2*ν*_*λτ*_*ν*_*τλ*_−*ν*_*λz*_)^−1^
	*C*_*λτλτ*_ = *G*_*λτ*_
Young moduli	${E}_{\lambda }=[{E}_{{\rm{A}}}{t}_{{\rm{A}}}{\mathrm{(1}-{\nu }_{{\rm{A}}}^{2})}^{-1}+{E}_{{\rm{B}}}{t}_{{\rm{B}}}{\mathrm{(1}-{\nu }_{{\rm{B}}}^{2})}^{-1}\mathrm{](1}-{\nu }_{\lambda z}^{2})$
	*E*_*τ*_ = [(1−2*ν*_A_)(1 + *ν*_A_)*t*_A_(*E*_A_(1−*ν*_A_))^−1^ …
	$${+\mathrm{(1}-2{\nu }_{{\rm{B}}}\mathrm{)(1}+{\nu }_{{\rm{B}}}){t}_{{\rm{B}}}{({E}_{{\rm{B}}}\mathrm{(1}-{\nu }_{{\rm{B}}}))}^{-1}+2{\nu }_{\lambda \tau }^{2}{({E}_{\lambda }\mathrm{(1}-{\nu }_{\lambda z}))}^{-1}]}^{-1}$$
Poisson ratii	${\nu }_{\lambda z}=[{E}_{{\rm{A}}}{t}_{{\rm{A}}}{\nu }_{{\rm{A}}}{\mathrm{(1}-{\nu }_{{\rm{A}}}^{2})}^{-1}+{E}_{{\rm{B}}}{t}_{{\rm{B}}}{\nu }_{{\rm{B}}}{\mathrm{(1}-{\nu }_{{\rm{B}}}^{2})}^{-1}][{E}_{{\rm{A}}}{t}_{{\rm{A}}}{\mathrm{(1}-{\nu }_{{\rm{A}}}^{2})}^{-1}+{E}_{{\rm{B}}}{t}_{{\rm{B}}}{\mathrm{(1}-{\nu }_{{\rm{B}}}^{2})}^{-1}{]}^{-1}$
	*ν*_*λτ*_ = [*ν*_A_*t*_A_(1−*ν*_A_)^−1^ + *ν*_B_*t*_B_(1−*ν*_B_)^−1^](1−*ν*_*λz*_)
	$${\nu }_{\tau \lambda }={\nu }_{\lambda \tau }{E}_{\tau }{E}_{\lambda }^{-1}$$
Shear modulus	*G*_*λτ*_ = *G*_A_*G*_B_(*t*_A_*G*_B_ + *t*_B_*G*_A_)^−1^
Thermal expansion	${\alpha }_{\lambda \lambda }=[{\alpha }_{{\rm{A}}}{E}_{{\rm{A}}}{t}_{{\rm{A}}}{\mathrm{(1}-{\nu }_{{\rm{A}}})}^{-1}+{\alpha }_{{\rm{B}}}{E}_{{\rm{B}}}{t}_{{\rm{B}}}{\mathrm{(1}-{\nu }_{{\rm{A}}})}^{-1}\mathrm{](1}-{\nu }_{\lambda z}){E}_{\lambda }^{-1}$
	*α*_ττ_ = *α*_A_*t*_A_(1 + *ν*_A_)(1−*ν*_A_)^−1^ + *α*_B_*t*_B_(1 + *ν*_B_)(1−*ν*_B_)^−1^−2_νλ_*τα*_λλ_(1−*ν*_λz_)^−1^

## Results

**Figure 2:**
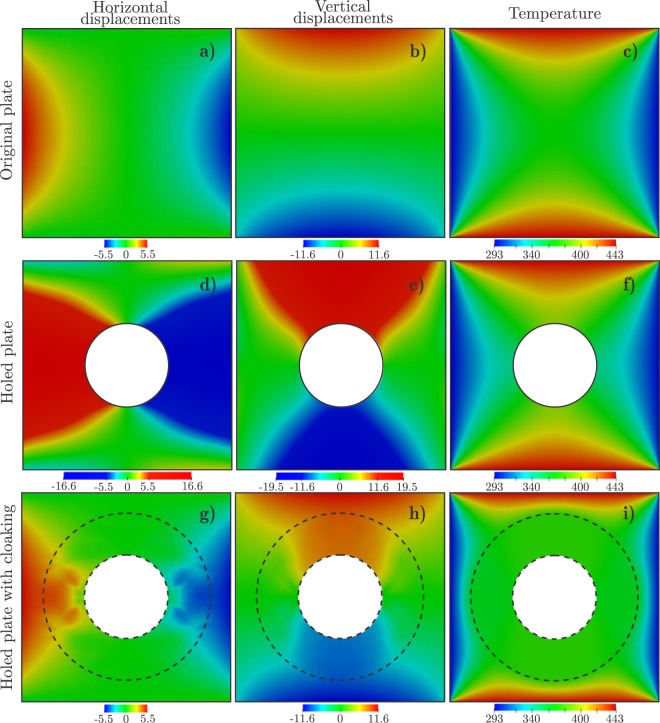
Displacements and temperature distributions for the homogeneous plate without hole, the homogeneous plate with hole, and the plate with the cloaked hole. Displacements and temperature distributions are given in millimeters and Kelvin, respectively.

Considering the nylon plate without the hole, the FEM solution for the displacement and temperature fields, that is **u**_0_ and *T*_0_, are that given in Fig. 2a–c; note that ${\rm{\max }}\Vert {{\bf{u}}}_{0}\Vert =11.63\,{\rm{mm}}$. For the plate with the hole, we use the mesh of 35053 square bilinear finite elements obtained by simply discarding the elements whose centers lie in Ω_incl_. Without a cloaking device, the displacement and temperature fields in the holed plate are that shown in Fig. 2d–f, and the error in the accomplishment of the cloaking task is ${{\rm{RMSE}}}_{0}=6.18\,{\rm{mm}}=0.53\,{\rm{\max }}\Vert {{\bf{u}}}_{0}\Vert$.

For the cloaking problem, the 15086 elements of the holed mesh whose centers lei in Ω_met_ are assumed to have variable microstructure (to be determined), whereas the remaining elements (those lying outside Ω_met_) are still made of nylon. The microparameters *t*^(*e*)^ and *θ*^(*e*)^ of the former make the design variable vector **P**, whereas the centers of the latter are the checkpoints for the cloaking task accomplishment. Note that **P** has dimension dim**P** = 2 × 15086 = 30172. Further, each admissible *P*_*i*_ must lie in the interval [0, 1], which is a bound constraint. Like in standard material distribution problems^36^, the checkerboard-type instabilities are prevented using a linear density filter^37^ identically as done by Peralta *et al*.^10^ for the design of purely thermal metamaterials.

**Figure 3:**
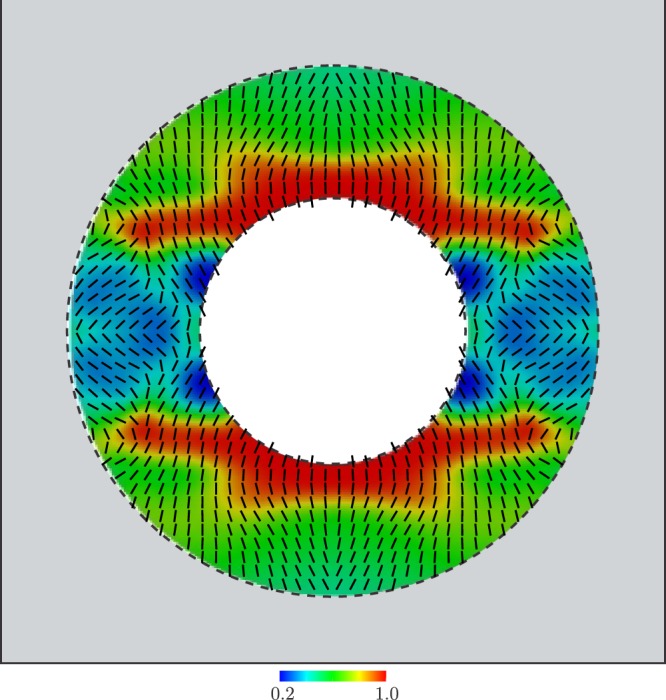
Variable microstructure computed by the solution of the nonlinear large-scale optimization problem.

Finally, the nonlinear large-scale constrained optimization problem given by equation (4) is solved using the method of moving asymptotes (MMA)^38^. The so-computed variable microstructure is shown in Fig. 3, where the relative thickness of aluminum and the orientation are represented by a colormap and oriented segments, respectively. The displacement and temperature fields computed by including a cloaking device with such a microstructure, are shown in Fig. 2g–i. Quantitatively, using this optimal device, the cloaking task is achieved up to an error RMSE = 0.119 mm = 0.0192 RMSE_0_.

### Extension to multiple thermal and mechanical boundary conditions

In order to prove the versatility of the methodology proposed in this communication, let us examine the case of cloaking under multiple thermal and mechanical boundary conditions. For this purpose, let us consider a straightforward redefinition of the objective function to be minimized. Let ${{\bf{u}}}_{0}({\bf{x}},{ {\mathcal B} }^{(\alpha )})$ be the displacement under the set of thermal and mechanical boundary conditions ${ {\mathcal B} }^{(\alpha )}$ in the domain Ω without the inclusion, and ${\bf{u}}({\bf{x}},{\bf{P}},{ {\mathcal B} }^{(\alpha )})$ be the displacement under that set of boundary conditions in the presence of the inclusion Ω_incl_ together with the cloaking metamaterial occupying Ω_met_, where the effective thermal and mechanical properties throughout the metamaterial are defined by **P**.

In order to account for multiple boundary conditions ${ {\mathcal B} }^{\mathrm{(1)}},{ {\mathcal B} }^{\mathrm{(2)}},\ldots ,{ {\mathcal B} }^{({N}_{{\rm{set}}})}$, the minimization problem must be defined in terms of the weighted sum of the RMSEs in the cloaking task accomplishment under each set of boundary conditions ${ {\mathcal B} }^{(\alpha )}$:

5 $$\mathop{{\rm{\min }}}\limits_{{\bf{P}}\in {\mathscr{D}}}\sum _{\alpha =1}^{{N}_{{\rm{set}}}}\,{\omega }_{\alpha }\sqrt{\frac{1}{{N}_{{\rm{check}}}}\sum _{i=1}^{{N}_{{\rm{check}}}}{\Vert {\bf{u}}({\bar{{\bf{x}}}}^{(i)},{\bf{P}},{ {\mathcal B} }^{(\alpha )})-{{\bf{u}}}_{0}({\bar{{\bf{x}}}}^{(i)},{ {\mathcal B} }^{(\alpha )})\Vert }^{2}},$$

where *ω*_*α*_ is the weight assigned to the task accomplishment under the boundary conditions ${ {\mathcal B} }^{(\alpha )}$.

**Figure 4:**
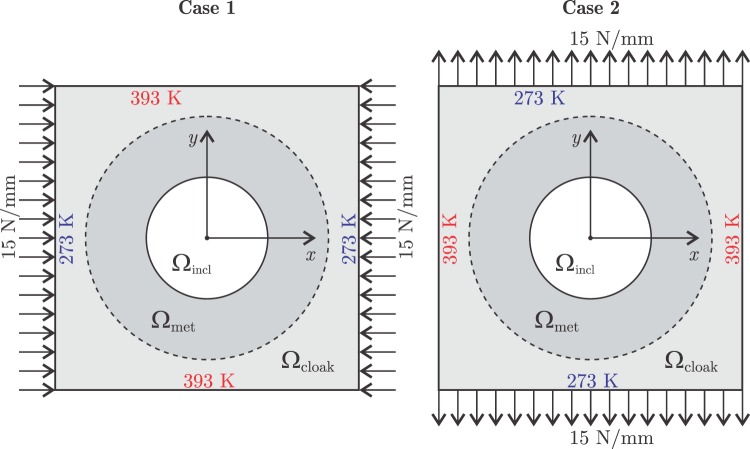
Domain Ω = Ω_cloak_ ∪ Ω_met_ ∪ Ω_incl_ of the thermo-mechanical problem extended to multiple boundary conditions.

**Figure 5:**
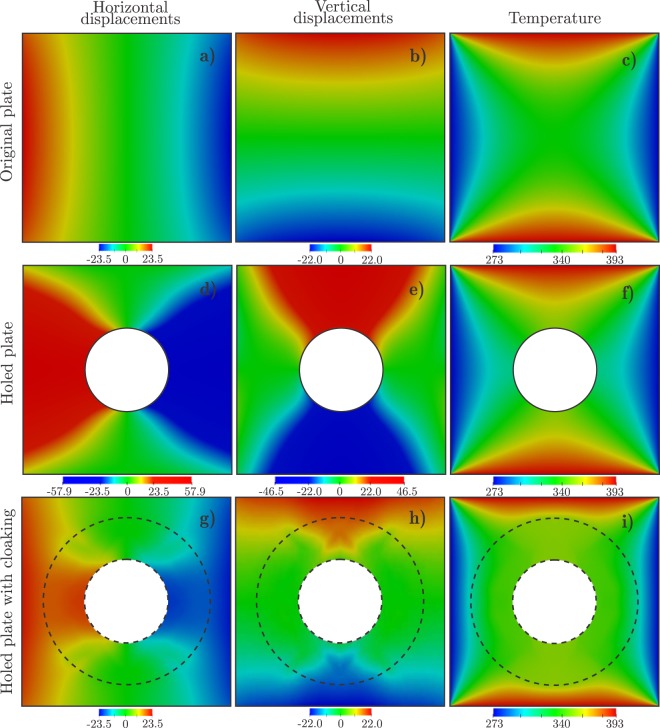
Thermo-mechanical problem extended to multiple boundary conditions. Displacements and temperature distributions for the homogeneous plate without hole, the homogeneous plate with hole, and the plate with the cloaked hole, under the boundary conditions of Case 1. Displacements and temperature distributions are given in millimeters and Kelvin, respectively.

**Figure 6:**
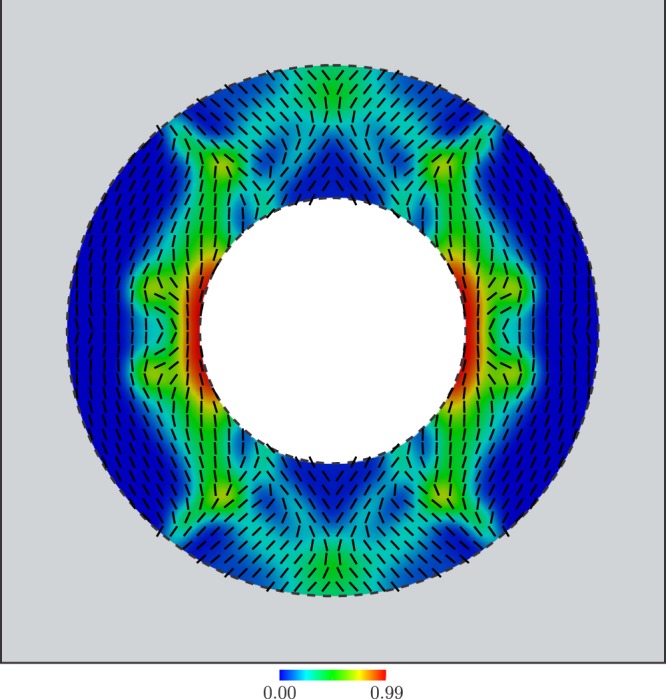
Thermo-mechanical problem extended to multiple boundary conditions. Variable microstructure computed by the solution of the nonlinear large-scale optimization problem.

As an applied example, let us consider the design of a single device for the fulfillment of the cloaking task under two different sets of thermal and mechanical boundary conditions, just as depicted in the cases 1 and 2 of Fig. 4, respectively. The domain dimensions remain the same as depicted in Fig. 1. The optimization-based procedure proposed in this study can also be conducted including the dependence of the mechanical properties with temperature. In this extended version of the thermo-mechanical cloaking problem, the metamaterial will be designed including the dependence of Young’s modulus on temperature. The Young’s modulus temperature-dependent functions of the background material (nylon) and the constituent materials of the laminate (aluminum and polyethylene) have been obtained from a non-linear regression of the data reported in the references of Table 3. The remaining materials properties are kept constant, with the values reported in Table 1. However, this methodology allows the possibility of introducing the temperature dependence of any property without further complications. It should be noted that even if such properties are constant for each constituent material of the laminate, the single dependency of the Young’s modulus with temperature introduces a change in all the effective mechanical properties listed in Table 2. Without a cloaking device, the error in the accomplishment of the extended cloaking task is RMSE_0_ = 11.325 mm, with ${{\rm{RMSE}}}_{0}^{(1)}=19.23\,{\rm{mm}}=0.75\,{\rm{\max }}\Vert {{\bf{u}}}_{0}^{(1)}\Vert$ and ${{\rm{RMSE}}}_{0}^{(2)}=3.42\,{\rm{mm}}=0.29\,{\rm{\max }}\Vert {{\bf{u}}}_{0}^{(2)}\Vert$, for the boundary conditions of cases 1 and 2, respectively. The displacement and temperature fields in the nylon full plate and the nylon holed plate under the boundary conditions of Case 1, are shown in Fig. 5(a–f), respectively. The variable microstructure of the device designed to fulfill the global cloaking task is shown in Fig. 6.

**Table 3:** Dependence of the Young’s modulus of nylon, aluminum and polyethylene with temperature.

Nylon^39^	Aluminum^40^	Polyethylene^41^
2.334 [1.208−(1 + *e*^−0.141*T* + 48.5^)^−1^] GPa	*E*_A_ = −0.04 *T* + 80 GPa	*E*_B_ = 136.155 *e*^−0.0161*T*^ GPa

The displacement and temperature fields computed by including a cloaking device with such a microstructure, are shown in Fig. 5(g–i). Using this optimal device, the cloaking task is achieved up to an error RMSE = 1.535 mm = 0.136 RMSE_0_, with ${{\rm{RMSE}}}^{(1)}=2.52\,{\rm{mm}}=0.131\,{{\rm{RMSE}}}_{0}^{(1)}$ and ${{\rm{RMSE}}}^{(2)}=0.55\,{\rm{mm}}=0.161\,{{\rm{RMSE}}}_{0}^{(2)}$. Such results demonstrate the possibility of designing a metamaterial to accomplish a thermo-mechanical cloaking task under multiple boundary conditions, by means of the optimization-based procedure proposed in this work. Also, it is worth noting that a device designed for multiple boundary conditions performs the cloaking task for each individual case poorly when compared with the device specifically designed for one of them, as done in the previous section.

In conclusion, we introduced the design of thermo-mechanical metamaterials, which is an unprecedented topic in the literature, using the optimization-based approach. With the task of elastostatic cloaking under thermal loads as an example, any task measured by an objective function depending on temperature and displacements (extreme thermal expansion, for instance) can be accounted for. This method allows the fulfillment of such a task, if not exactly, optimally, using realizable metamaterials. Actually, it dictates how to fabricate the metamaterial. To avoid the need of simultaneously mimicking several thermal and mechanical properties, which is highly uncertain, is a crucial advantage of this novel approach with respect to the TO procedures (in case they are to be used for thermo-mechanical metamaterials). Although the realizability of the so-determined microstructure is assured, we will evaluate strategies for the design of easier-to-fabricate thermomechanical devices, following our developments for thermal^11,12^ and mechanical metadevices^16^, where piecewise homogeneous either isotropic^12,16^ or anisotropic^11^ materials were used. Thus, in future works, we plan to introduce such constraints in order to facilitate the fabrication of thermo-mechanical metadevices, taking into account the capabilities of polymer and metal 3D printers.
